# An Enhancement for IEEE 802.11p to Provision Quality of Service with Context Aware Channel Access for the Forward Collision Avoidance Application in Vehicular Ad Hoc Network

**DOI:** 10.3390/s21206937

**Published:** 2021-10-19

**Authors:** Tripti C, Jibukumar M G

**Affiliations:** 1 Department of Computer Science & Engineering, Rajagiri School of Engineering and Technology, Cochin 682022, India; 2 Division of Electronics, School of Engineering, Cochin University of Science and Technology, Cochin 682022, India; jibukumar@cusat.ac.in

**Keywords:** vehicular ad hoc network, co-operative collision warning, medium access control protocol, IEEE 802.11p, forward collision warning, wireless communication

## Abstract

Key application of an intelligent transportation system is traffic safety, and it provides driver assistance. Safety messages are of two types, beacon messages and event messages. The nodes broadcast these messages in the vehicular networks. The system must rely on a robust medium access control (MAC) protocol to support delivery of safety messages. The standard medium access scheme that is used in vehicular networks to provide service differentiation to support various applications is IEEE 802.11p. The emergency event messages should reach the drivers immediately to take necessary steps to avoid casualties on the road. In IEEE 802.11p, both of these messages are considered with the same priority so that no separate differentiation is created. The proposed work focuses on improving the quality of service for forward collision warning applications in intelligent transportation systems. The scheme proposes a priority-based cooperative MAC (PCMAC) for channel access that works on the context of information. Simulation and analytical results validate improved performance of PCMAC in terms of packet delivery ratio, throughput, and average packet delivery delay, as compared with other eminent MAC protocols. The simulation results show that it has a 9% higher improvement in throughput than IEEE 802.11p and has better performance in the increasing number of emergency messages.

## 1. Introduction

**Figure 1:**
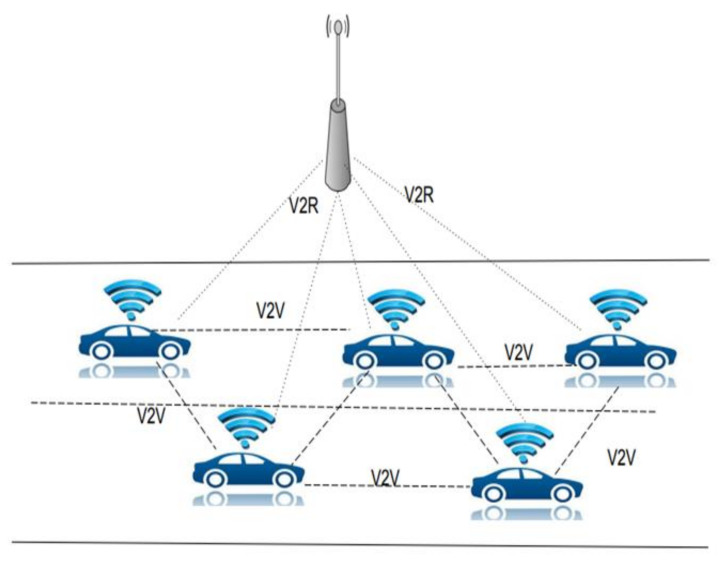
Structure of VANET.

Vehicular ad hoc network (VANET) has a major role in the development of an intelligent transportation system (ITS). It is defined as a wireless network that uses wireless local area network (WLAN) technology for vehicles on the road as the nodes of the network. The network supports communication between vehicles as well as between the vehicle and road-side unit. The VANET structure is depicted in Figure 1. The applications in VANET include traffic management, safety, and entertainment applications to improve the comfort and safety of road travelers.

The network topology changes often due to the high mobility of vehicles on the road [1]. This complicates the design of VANET resource allocation and routing. To support communication in VANET, the standard organization IEEE developed dedicated short-range communications (DSRC). VANET uses a frequency spectrum of 5.850 to 5.925 GHz [2]. This frequency group has seven channels, including a control channel (CCH) for the broadcast of control messages and six data-service channels to support VANET comfort applications.

**Figure 2:**
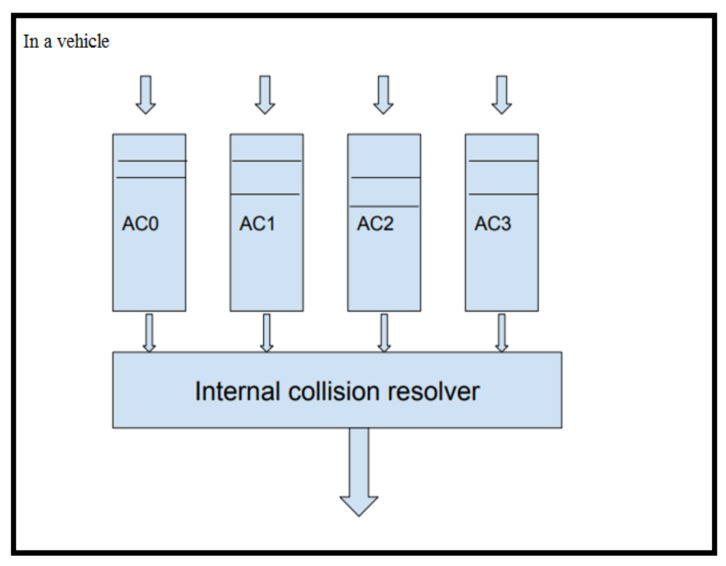
Working of IEEE 802.11p.

VANET follows IEEE 802.11p for medium access control. Later, the standard was amended, and VANETs began to use wireless access vehicular environment (WAVE) for security and operations with multiple channels. IEEE 802.11p follows an enhanced distributed coordination function (EDCF) for the access of the channel. EDCF is a contention-based method. Figure 2 shows IEEE 802.11p working in a vehicle. It categorizes the traffic in a vehicle into four based on the access category. The packets are scheduled internally by an internal scheduler. The scheduled packet from each node will have to contend with other nodes that follow the same protocol in the network for obtaining channel access for transmission.

The carrier sense multiple access/collision avoidance (CSMA/CA) technique is used by the nodes to access the channel. Before attempting transmission, the node will check the type of access category and adapt the transmission parameters as shown in Table 1. Then, the node will sense the channel for an arbitrary inter frame space (AIFS) period. If the transmission medium is idle for AIFS time, the node will select a back off value from the contention window at random, which will prevent a node from accessing the channel at the same time as other nodes. The node will continue sensing the medium during the back off period. If the medium is not free, the back off timer will be frozen, and the node will try to connect the channel after waiting for some time. If the medium is free, the node will access the channel. IEEE 802.11p follows the broadcast mode of communication to transmit safety messages over the control channel. The transmission is not acknowledged, as too many acknowledgements from the nodes can congest the channel. IEEE 802.11p has a poor performance for safety messages [3].

### 1.1. Motivation and Objectives

The main application in the Intelligent Transportation System is the provision of driver assistance to avoid casualties on the road. The forward collision warning application of co-operative collision warning works with the safety messages. The safety messages are of two types, and the first one is the routine safety messages (non-emergency) that are periodically updated by the nodes. These messages contain the details of its position, speed, etc. The second type of safety message is the event safety (emergency) messages that occur due to certain events that happen on the road. Safety messages are disseminated through the control channel to the neighboring nodes by means of broadcast mode of communication. Therefore, they follow AIFSN with two and a contention window range of three. These messages must be disseminated quickly so as to make the driver take the necessary steps to avoid accidents. However, in IEEE 802.11p, both these safety messages are considered with the same priority, and broadcast messages lack an acknowledgement. Therefore, there is a chance that the messages may not reach the neighboring nodes on time.

Packets from an emergency vehicle should be transmitted with priority as its impact on other vehicles is high. The routine safety messages also must be given a fair chance to be transmitted, as it is necessary for the vehicles to be alerted about the details of neighbor nodes to avoid accidents. In this work, a priority-based cooperative MAC that works with an adaptive contention window mechanism for the provision quality of service in the forward collision warning application is proposed. The scheme studies the context of a packet, its average relative distance with other nodes, and the average velocity of the platoon to calculate the priority of the packet. The priority values of the packet from on-the-road cars are used to adaptively modify the contention window parameter.

### 1.2. Novelty and Contributions

We proposed a priority-based cooperative medium access control (PCMAC) protocol that makes the following contributions:

1. The protocol improves IEEE 802.11p performance for supporting forward collision warning applications in VANET. The scheme improves the delivery of emergency (event) messages by prioritizing them over the non-emergency messages by using an adaptive contention window.
2. The proposed scheme provides a service differentiation among the emergency and non-emergency message in the safety category of IEEE 802.11p by implementing a dynamic contention window for the emergency packets based on the priority of the packet from the node at the instant. The non-emergency message, the routine safety message in IEEE 802.11p, follows the same strategy that was implemented in IEEE 802.11p.
3. The performance of the broadcast with service differentiation for emergency messages over non-emergency message in the safety category of IEEE 802.11p with an analytical model is studied.

### 1.3. Paper Organization

The remaining section of the paper is structured as follows. Related research reported in the relevant area is outlined in Section 2. Section 3 describes the proposed priority-based cooperative MAC. Section 4 presents the mathematical modeling of the scheme, followed by discussions on the results obtained in Section 5. Section 6 gives the concluding remarks.

## 2. Related Work

**Figure 3:**
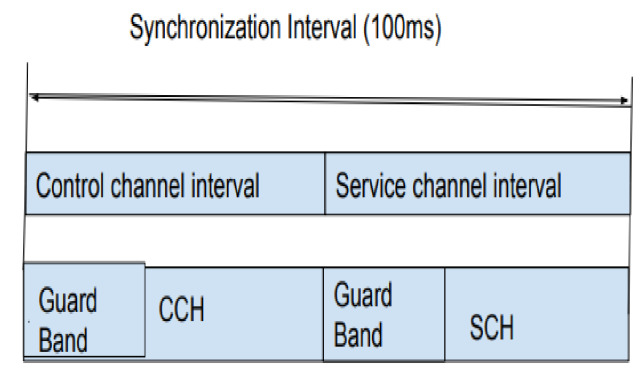
Synchronization interval [5].

According to the literature, the idea of Vehicular Ad-hoc Networks was introduced in the early 1980s, and research in this field exploded in the 2000s. The vehicles use the channels in the communication band (5.8–5.9 GHZ) designated by DSRC to convey non-safety and safety messages. DSRC also developed IEEE 802.11p as the MAC for resource sharing. Later, with the amendment of wireless access in vehicular environments (WAVE), the system moved on to a multi-channel scenario that follows a synchronous interval that alternates between control channels and data channels, as shown in Figure 3. VANET broadcasts control messages over the control channel and data services over the service channels. The safety messages and data channel negotiation happen at the control channel and during the service channel interval; non-safety data transfer happens at the negotiated data channel. Each vehicle must compete with other cars for the control channel to convey data. The priority of safety communications is always higher than that of non-safety data communications. The control channel communication happens by broadcast. The broadcast message will not have an acknowledgement from the receivers. When the node density increases, the contention for channel increases, and as a result, the chances of the message to be transmitted are low. The studies show that IEEE 802.11p has a poor performance with broadcast [3].

The safety messages can be both the routine update messages that are periodically sent between vehicles [6] and the event messages that are created upon the occurrence of an event [7]. IEEE 802.11p supports service differentiation for different applications in VANET. Many studies have been conducted to enhance the performance of IEEE802.11p by modifying the transmission parameters [8,9,10,11]. Bianchi suggested a statistical approach to analyze the channel utilization and throughput [12]. In this work, safety message transmission assures the quality of service by improving the performance of IEEE 802.11p for forward collision avoidance.

The transmission parameter and contention window is modified to improve the performance of IEEE 802.11p, adapting it to various applications of VANET. Existing works that alter the contention window with cooperative collision warning that supports QoS with broadcast communication is studied in this section.

To meet the needs of the IEEE802.11p network with fluctuating network density in [13], the minimum and maximum contention windows are changed based on network performance. The authors in [14] discuss dynamic contention window adaption and transmission power for automobiles on the basis of the density of nodes and the packet collision rate. However, the performance of the scheme will be affected at high power levels. The authors [15] use the traffic density in the network to alter the contention window size. The performance of IEEE802.11p with an adaptive back-off window is examined in two different ways in [16]. The first is a centralized method in which the base station calculates an appropriate contention window based on vehicle density. The second technique is a distributed approach, in which each vehicle chooses the back-off time based on their local channel information. A self-adjusting contention window-based technique for increasing VANET efficiency that utilizes the persistence factor is discussed in [17]. The authors of [18] discuss an adaptive contention window approach that maps the priority value to the node density.

The method can effectively increase overall throughput and reduce packet collision rate and delay time. However, the contention windows in these schemes are calculated as the function of the sum of the number of competing nodes detected by both the sender and the receiver. The number of competing nodes vary at each instant, and it is difficult to track this value. The schemes do not consider the mobility of the nodes.

IEEE 802.11p provides service differentiation for different categories of data. However, it treats the beacon and event message as a safety category. The event messages should be given higher priority when compared to the beacon messages. The drivers can react as they receive the event messages and thereby reduce the number of accident cases on the road. The beacon message is periodically broadcast. It should also be given a fair chance to access the channel, as it is very much needed by the vehicles to learn about the neighboring vehicles in its locality. Many literatures have priority assignment in combination with IEEE802.11e. In [19], an inter-vehicle communication, where the chance of transmission is improved by repeatedly delivering high priority signals over low priority messages, is studied. However, there are chances for the high priority signals to occupy the channel always, and this leads to the under-utilization of service channels. The authors of [20] concentrated on the issue of ensuring fairness for the nodes with varying mobility under each RSU. The topic of balancing throughput and fairness is treated as a multi-objective optimization problem in [21]. RSU calculates the ideal transmission probability, and the nodes begin transmission at a lower level than this value. It calculates the fairness index after a brief period and dynamically updates the probability of transmission based on the value. However, the threshold for the probability of transmission is set per the ideal channel conditions, and this may vary the performance. In [22], the velocity and density of the network is considered to provision the QoS of different traffic classes. However, these schemes do not consider the network conditions along with the velocity of the node, which plays a pivotal role in performance of the system.

In [23], the suitability of DSRC to support vehicular safety application called cooperative collision warning (CCW) is studied. It also discusses the different types of applications that are issued under CCW. They found that the necessary QoS is dependent upon the application’s parameters setting. In [24], a rapport between network traffic and emergency cases over the highway is studied. The article studied some of the emergency cases over the highway and showed that the impact of accidents on the near-by vehicles traveling is grounded on parameters such as speed, relative location of the nodes from the accident, and moving direction of the vehicle. The work aims to improve throughput for the reduction of travel time, fuel consumption, etc. In [25], the focus is on achieving low latency for delivering emergency warnings in case of an event. The authors discuss the application challenges, focusing on congestion control issues linked with the vehicular cooperative collision warning application. It considers the existence of one or more broken vehicles. The simulation results showed that the warning message delivery delay is low when the channel conditions are poor. In [26], priority-based direction aware MAC protocol is discussed. The protocol proposes a cluster based V2V MAC protocol called PDMAC for prioritizing warning message delivery in VANETs. It considers the severity level, direction, and message type. PDMAC also suggests a method to synchronize the clock for inter-vehicular communication. The scenario considered in this work is a highway. It reduces message loss rate and end-to-end delays, increasing network throughput. However, the protocol has communication overhead. It considers only the speed factor to analyze the severity of the message. In [27], a frame in this protocol has two types of slots, TDMA slots for beacon broadcast and CSMA slots for transmitting warning messages. It uses a stochastic model based on minimum safety distance to forecast the average number of accidents that could happen in the platoon. This value is used to determine the part of CSMA segment in the time frame of the protocol. However, the transmission delay is relatively larger in an overload traffic scenario.

Broadcast over the control channel is studied using an analytical model in [28]. V2V collision avoidance applications with context-aware communication is studied in [29]. The authors of [30] discuss the prioritization of received data flow on the basis of the impact of the message on a vehicle acting as receiver. This paper states that prioritization must be done based on severity metric. The work defines severity based on the application as a metric of vehicle speed, direction, and position. A dynamic information quantity-based to an emergency-degree is studied in [31] to support safety service in VANET. The work deals with performance improvement of the broadcast in IEEE 802.11p using dynamic updating of contention window based on the change in vehicle density.

The studies on the safety message dissemination can be classified into three categories: (1) those based on network statistics and density, (2) those based on the mobility of nodes, and (3) those based on the severity of the message. The last category combines the first two categories with other parameters such as the direction, location, and access type to learn the severity of the message. The overview of the works is summarized in Table 2.

The review of the literature clearly demonstrated the necessity for an enhancement in IEEE802.11p for the provision quality of service for the forward collision warning system. The proposed improvement is discussed in the next section.

## 3. Proposed Method: Priority-Based Cooperative MAC (PCMAC)

The proposed work presents a strategy for improving IEEE 802.11p for the forward collision warning application in VANET. The scheme tries to dynamically update the contention window so as to achieve service differentiation between emergency and non-emergency messages in the safety message category of IEEE 802.11p.

### 3.1. Relevance of the Proposal

As discussed above, vehicular ad hoc networks (VANET) have several applications, from traveler comforts to life saving accident prevention schemes. *Lifesaving applications* attract more attention due to the casualties reported by road accidents every year. In VANET, nodes learn and compute collision probabilities between them at regular intervals by means of cooperative collision avoidance schemes. Based on these probabilities, warning/emergency messages used to alert the nodes to take preventive measures are communicated to the nodes either directly or through the road-side unit [32].

Apart from identifying the possibility of collision among nodes, in–time delivery of emergency messages is important in cooperative collision avoidance schemes. This is because the driver needs to take preventive measures before an accident occurs. Emergency message dissemination is handled in both networks and MAC layers. This paper focuses on the MAC layer. The MAC layer is associated with the sharing of medium. In this layer, VANET uses IEEE 802.11p that follows carrier sense multiple access (CSMA/CA) as the channel access standard for communication between the nodes. It helps in disseminating the message without waiting for a reserved slot as in TDMA. However, as the number of nodes in the region increases, it shows a low performance [8]. This points to the need for an efficient channel access scheme and message delivery at the MAC layer. Giving equal priority to all the nodes will limit the performance of the system. This will delay the delivery of emergency messages of high accident probability nodes. An approach that prioritizes the delivery of emergency messages is the assignment of a higher priority to emergency messages over non-emergency messages. The aim of this paper is to improve the performance of IEEE 802.11p, the traditional MAC, and ensure timely delivery of emergency messages to avoid successive collision in VANET. The proposed protocol is an extension of the CSMA/CA approach. Our system is compatible with the existing standards, aiming to have low latency in the dissemination of emergency messages compared to IEEE 802.11p. In IEEE 802.11p, both beacon and safety messages are considered to have the same priority. In this protocol, the system differentiates the warning messages from the periodic beacons by prioritizing the message with respect to its severity.

### 3.2. Scenario

**Figure 4:**
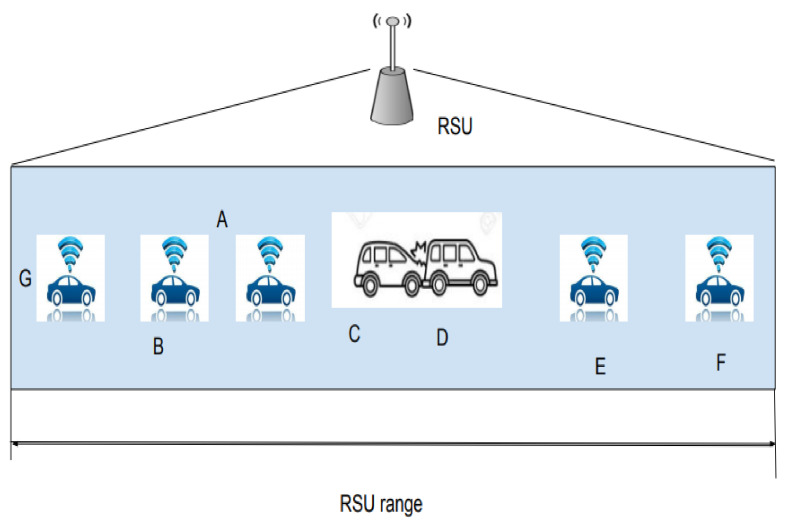
Scenario.

**Figure 5:**
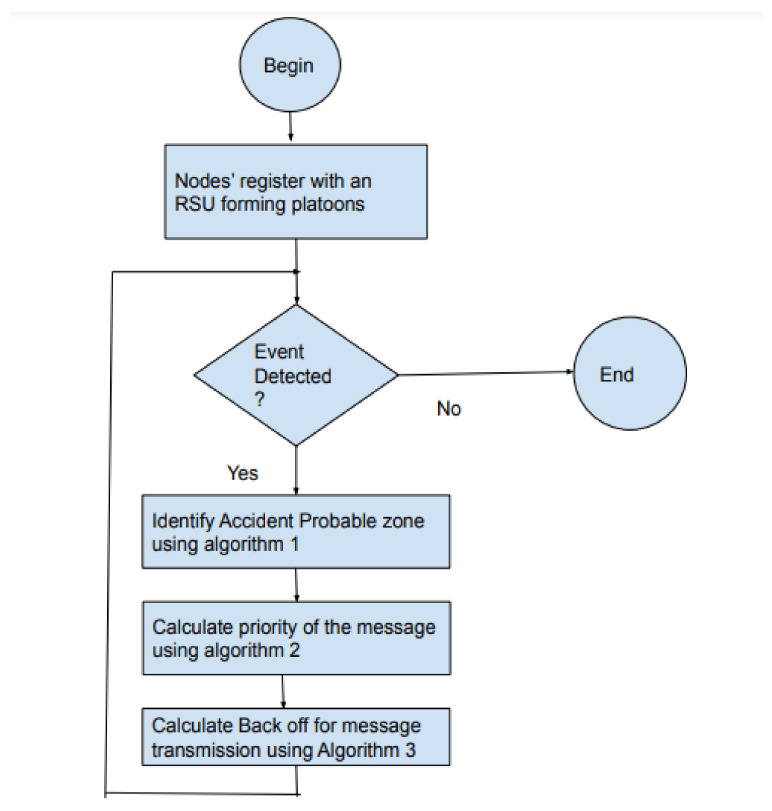
Overview of the proposed priority-based cooperative MAC (PCMAC) protocol.

The scenario considered is depicted in Figure 4. The system model considers a platoon of cars traveling in a single lane in the same direction. Each vehicle knows its location and velocity at that instant. In this work, we take the assumption that every RSU can communicate with vehicles within its coverage, and so the RSU can compute the vehicle density within its coverage. Suppose that the car at the leading side stops abruptly due to some unexpected event, the vehicles behind should be notified quickly so that they can apply the break and avoid successive collision. The popular scheme to avoid forward collisions is to use brake lights to warn the vehicles behind. However, there are many challenges in implementing this [23]. The forward collision happens when the drivers behind the vehicle that abruptly stopped are not aware of the event that has happened, and they are not able to control their vehicle speed, ending up in a successive rear end collision. In the scenario discussed, a driver can see only the brake lights from the vehicle in front of them. The vehicle A can see the event and brake lights, but the vehicles B and G will not know the emergency at C until vehicle A breaks. Another reason is the mobility of the vehicles. Due to mobility, the signal is irrelevant for vehicles E and F. The neighbor nodes of the vehicles differ due to mobility. An overview of the proposed protocol is presented in Figure 5. The methodology starts with the registration of vehicles under an RSU. The vehicles that come within range of the RSU register with the RSU. At the time of registration, the node is assigned an ID along with the current capacity of the nodes in its region.

**Figure 6:**
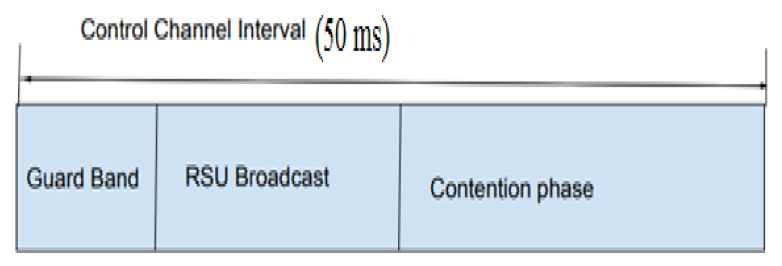
Time Frame.

**Figure 7:**
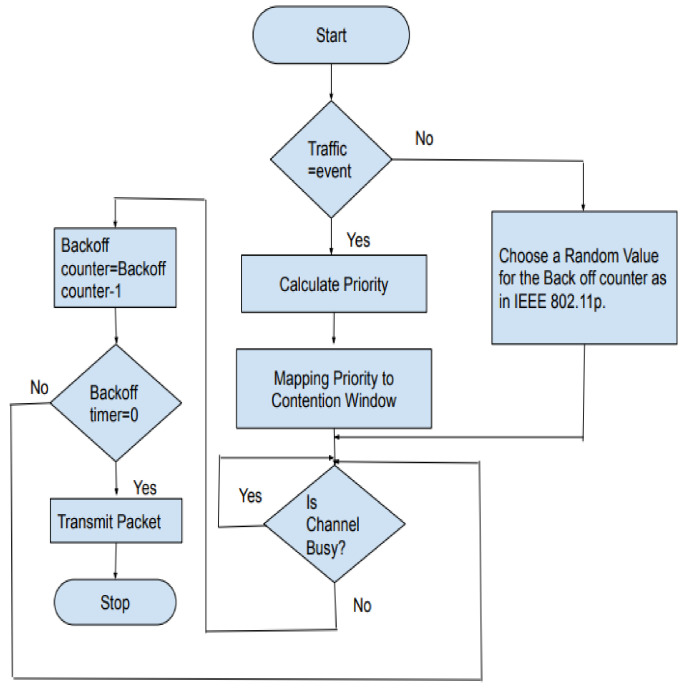
Flow of execution of the proposed protocol.

The RSU (road-side unit) in the scenario communicates with the vehicles and frames to the vehicles at periodic intervals. The time frame used in the protocol is shown in Figure 6. During the first phase, RSU will broadcast the details of the registered vehicles to the platoon. It includes the vehicle IDs, the number of vehicles registered in range, and the average velocity of the platoon. The protocol needs to provide the nodes with access to the channel for the transmitting of both beacon and event messages. In the control channel, the nodes will first listen to the road-side unit. In the second phase, PCMAC follows a random-access mechanism that selects a back-off value from the contention window depending on the priority of the packet at that instant. The flow of execution of the protocol is as shown in Figure 7.

The notations used in the protocol are represented in Table 3.

The vehicles follow the IEEE 802.11p contention window for non-emergency messages, and priority-based channel access is used for emergency messages. The priority calculation and priority mapping are introduced in the existing EDCA scheme for the development of the protocol to support timely delivery of emergency messages. These two blocks will be explained in the following section.

### 3.3. Priority Calculation

The vehicles that follow the vehicle in the accident will be in danger. Therefore, we identify an accident zone initially, and within the accident zone the priority of the node is calculated. The accident zone is identified using Algorithm 1.
**Algorithm 1:** Algorithm to find the accident zone1: **procedure** ACCIDENT ZONE (x_s_(t), y_s_(t), *R*, *x_r_*, *y_r_*)// location of vehicle in accident, R-Range and Range coordinates2:  ds(t) = $\sqrt{{\left((x_{r}-x_{s}\left(t\right)\right)}^{2}+{\left(y_{r}-y_{s}\left(t\right)\right)}^{2})}$3:  **while** ds(t) ⇐ R/2 **do**4:    Read Accident Zone from $\left[(x_{s}\left(t\right),y_{s}\left(t\right),\;\left(x_{s}\left(t\right)-\frac{R}{2},y_{s}\left(t\right)-\frac{R}{2}\right)\right]$5:  **while** ds(t) > R/2 **do**6:    B(t) = R − ds(t); 7:    Get $B_{x}\left(t\right)$ and $B_{y}\left(t\right)$; 8:    Read Accident Zone from $\left[\left(x_{s}\left(t\right),y_{s}\left(t\right),B_{x}\left(t\right),B_{y}\left(t\right)\right)\right]$


The priority of a node at an instant depends on the importance of the packet on other vehicles. This is calculated based on three parameters. They are:

- *Impact_i_*(*t*): This defines the severity of the event. This paper mainly concentrates on the forward collision warning application. The forward collision happens when the vehicles behind are not able to control their speed as the vehicle in front stops abruptly. This can be avoided by quickly alerting the vehicles behind the broken vehicle so that the drivers can control the speed of their vehicle to avoid successive collision. The maximum number of collisions that can occur can be equated to the number of vehicles behind the vehicle that has entered a collision. *Impact_i_*(*t*) measures the severity of the accident. It is calculated as a function of the factor of the number of nodes that enter into the accident probable zone with the total number of nodes in the platoon. The vehicles close to the broken node have more chances to collide. Therefore, we consider a probable zone of accident occurrence. This zone is defined based on the location of the accident. If the vehicle in the accident has a distance to cover the range that is less than half the range, it will consider vehicles between its point and half of the range. Otherwise, it will look for the vehicles between the maximum range and its point. Algorithm 2 shows the steps to determine the accident probable zone. Equation (1) defines *Impact_i_*(*t*) of a vehicle at an instant *t.*
  (1) $$Impact_{i}\left(t\right)=\frac{Number\;of\;vehicles\;behind\;the\;vehicle_{i}\;at\;instant\;t}{Total\;number\;of\;vehicles\;in\;the\;platoon}$$
- *AD_i_*(*t*): The average relative distance of nodes with respect to node_i_ at instant *t*. The average relative distance of the vehicles with respect to the vehicle involved in the accident in the probable area of accident occurrence is considered. This can be represented as Equation (2). The nodes with a less than average relative distance will be more highly affected. The lesser the average relative distance, the higher the chance to have a forward collision.
  (2) $$AD_{i}\left(t\right)=\frac{1}{\left(N-1\right)}\overset{N}{\underset{i=1}{\sum }}D_{ij}\left(t\right)$$
- *AV*(*t*): The average velocity of the platoon at instant *t*. This can be represented as Equation (3). The platoon with a high average velocity will be affected more.
  (3) $$AV\left(t\right)=\frac{1}{N}\overset{N}{\underset{i=1}{\sum }}V_{i}\left(t\right)\;$$

The priority of an event packet from a node at an instant is calculated as given in Equation (4).

(4) $$Priority_{i}\left(t\right)=\frac{AD_{i}\left(t\right)}{Impact_{i}\left(t\right)*AV\left(t\right)}$$

**Algorithm 2:** Priority or an event message at a node for an instant t1: Read input: Time (t), Number or nodes (N(t)), position(x(t), y(t)). Type of traffic (e(t)), velocity (v(t))2: Calculate $Impact_{i}\left(t\right)$     // as shown in Equation (1)3: Calculate $AD_{i}\left(t\right)$       // as shown in Equation (2)4: Calculate $AV\left(t\right)$       // as shown in Equation (3)5: procedure *PRIORITY*(*Impact_i_*(*t*),*AD_i_*(*t*),*AV*(*t*)) 6: Calculate *Priority_i_*(*t*)     // as shown in Equation (4)

### 3.4. Priority Mapping to Contention Window

The priority value is used to select the contention window. The new maximum contention window is designed as shown in Equation (5).

The *CW_max_*[*i*] and *CW_min_*[*i*] is taken as (3) and (7), respectively. The access parameter contention window of the voice is taken for the calculation of the contention window for emergency messages.

(5) $$CW_{newmax}\left[i\right]=\frac{\left(P*10\right)*\left(CW_{max}\left[i\right]-CW_{min}\left[i\right]\right)}{13}$$

The back off timer is a random value between 0 and $CW_{newmax}$.

Algorithm 3 details the selection of a new maximum contention window mechanism used in the proposed protocol. The access parameter contention window of the voice category with *CW_min_* as (3) and *CW_max_* as (7) is taken as access parameters for non-emergency messages in the simulation.
**Algorithm 3:** This procedure calculates the Back-off value for a node1: **procedure** BACK·OFF(*traffic_i_*(*t*), *Priority_i_*(*t*), *CW_max_*, *CW_min_*)2: **while**
*traffic_i_*(*t*) == *emergency* do 3:  Calculate *CW_newmax_*(*t*) // as shown in Equation (5)4:  *Back-off_i_*(*t*) = *Random*([0,*CW_newmax_*(*t*)])5: **end while**6: **while**
*traffic_i_*(*t*) == *non-emergency*
**do**7:  *Back-off_i_*(*t*) = *Random*([*CW_min_*,*CW_max_*])8: **end while**9: **end procedure**

## 4. Mathematical Modeling

Safety message dissemination for driver assistance and comfort applications for travelers are the major applications in intelligent transportation systems. For supporting driver assistance, the vehicles will periodically broadcast safety messages to the other vehicles within its vicinity. However, as the vehicles density increases, the competition for channel access increases, leading to the deterioration of system performance. A message entering the buffer will be transmitted only if the previous message in the buffer is already sent. We have followed the model similar to [9,28]. These models used the sojourn time of the vehicle to model back-off. In PCMAC, the impact of a node or the severity of the node, as referred to in [30] with respect to an accident in its surrounding, is analyzed, and the nodes are prioritized based on their severity. The priority value of a node depends on the relative distance of the node from the accident, the average velocity of the platoon, and the position of the node at that instant. A one-dimensional Markov chain is used to study the performance of safety message broadcast in VANET.

**Figure 8:**
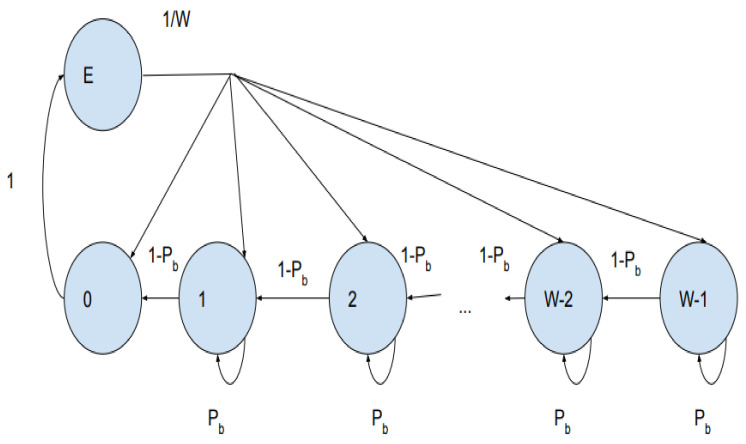
Markov chain model for broadcasting safety messages in VANET.

In Figure 8, P_b_ represents the chance of the medium to be busy and P_f_ = (1 − P_b_) represents the likelihood of the medium to be free. The values of P_b_ and P_f_ are defined in terms of CW, which is randomly chosen from the interval of 0 to (W−1). Vacant state of the buffer is represented as state E. After the transmission of a message, the packet will enter an empty state, so the transition probability to move from state 0 to state E is 1. The transition probabilities as per Figure 8 are as follows:

(6) $$\left\{\begin{array}{ll} P\left(j|j+1\right)=1-P_{b} & j\in \left[0,W-2\right]\\ P\left(j|E\right)=\frac{1}{W} & j\;\in \left[1,W-1\right]\\ P\left(j|j\right)=P_{b} & j\in \;\left[0,W-1\right]\\ P\left(E\;|0\right)=1 \end{array}\right.$$

Let ***b***(***t***) represent the stochastic processes for the back-off timer, and $b_{j}=\underset{t\to \infty }{\lim}P\left\{b\left(t\right)=j,\;j\in \left[0,W-1\right]\right\}$ can have the probability values of CW being k.

(7) $$\left\{\begin{array}{c} b_{0}=b_{E}\left(\frac{1}{W}\right)+b_{1}\left(1-P_{b}\right)\\ b_{j}=b_{E}\left(\frac{1}{W}\right)+b_{j+1}\left(1-P_{b}\right)+b_{j}P_{b}\;\;\;\;\;\;\;\;\;\;\;\;\;\;\;\;\;\;\;\;\;\;\;\;j\;\in \left[1,W-2\right]\\ b_{W-1}=b_{E}\left(\frac{1}{W}\right)+b_{W-1}P_{b} \end{array}\right.$$

Therefore, from (7) we can write as

(8) $$\left\{\begin{array}{c} b_{0}=b_{E}\\ b_{j}=\frac{b_{E}\left(W-j\right)}{W\left(1-P_{b}\right)}\;\;\;\;\;\;\;\;\;\;\;\;\;j\;\in \left[1,W-1\right] \end{array}\right.$$

(9) $$\overset{W-1}{\underset{j=0}{\sum }}b_{j}+b_{E}=1$$

After substituting (8) in (9) we obtain $b_{0}$. $b_{0}$ as the transmission probability of the node represented as τ.

(10) $$b_{0}=\tau =\frac{2\left(1-p_{b}\right)}{4\left(1-p_{b}\right)+\left(W-1\right)}$$

Equation (10) shows that the transmission probability depends on the contention Window (***W***).

The value of ***W*** is mapped to the priority of the node at an instant. Priority is calculated as a function of *Impact_i_*(*t*). The average relative distance of nodes is with respect to node_i_ at instant *t* and Average velocity of the platoon at instant *t*. Considering the range as 100 m and the maximum velocity of the vehicle as 100 Km/h, the maximum possible value of priority depends on the *Impact_i_*(*t*). Impact is measured as shown in Equation (1), where the maximum number of nodes possible behind a node is (N−1).

Let ***n*_0_** be the cardinality of the low priority node set and ***n*_1_** be the cardinality of the high priority node set. The collision probability of the beacon message is studied by analyzing the events that happen in time slots. In a time slot, the following events can happen:

- The probability of the medium to be idle, i.e., this condition happens when no node tries to occupy the channel.
  (11) $$P_{f}={\left(1-\tau_{0}\right)}^{n_{0}}{\left(1-\tau_{1}\right)}^{n_{1}}$$
- Probability of the channel to be busy, i.e., this condition happens when at least one node in the network tries to occupy the channel.
  (12) $$P_{b}=1-[{\left(1-\tau_{0}\right)}^{n_{0}}{\left(1-\tau_{1}\right)}^{n_{1}}]$$
- Probability of success, i.e., this condition happens when only one node selects the channel for transmission.
  (13) $$P_{s}=n_{0}\tau_{0}{\left(1-\tau_{0}\right)}^{n_{0}-1}{\left(1-\tau_{1}\right)}^{n_{1}}+n_{1}\tau_{1}{\left(1-\tau_{1}\right)}^{n_{1}-1}{\left(1-\tau_{0}\right)}^{n_{0}}$$

Therefore, the probability of collision, $P_{c}=1-P_{f}-P_{s}$.

(14) $$P_{c}=1-\left[{\left(1-\tau_{0}\right)}^{n_{0}}{\left(1-\tau_{1}\right)}^{n_{1}}\right]-\left[n_{0}\tau_{0}{\left(1-\tau_{0}\right)}^{n_{0}-1}{\left(1-\tau_{1}\right)}^{n_{1}}+n_{1}\tau_{1}{\left(1-\tau_{1}\right)}^{n_{1}-1}{\left(1-\tau_{0}\right)}^{n_{0}}\;\right]$$

PCMAC works with an adaptive contention window as per the priority of the packet from a node at an instant, and therefore, the expectation of back off value is given in Equation (15).

(15) $$E\left[B\right]=\overset{W-1}{\underset{i=0}{\sum }}\frac{i}{W}\left[\left(1-P_{b}\right)Slotvalue+P_{b}T\right]$$

In broadcast, the time of transmitting a packet,

(16) $$T=DIFS+T_{header}+T_{packet\;}$$

where $T_{header}=\frac{Header\;length}{Data\;rate}$ and $T_{packet}=\frac{Length\;of\;the\;packet}{Data\;rate}$.

For a broadcast operation, an acknowledgement is not obtained from the nodes to know whether the attempted transmission had a packet collision or successful transmission. Therefore, the time for a successful transmission $T_{success}$ and the time for a packet collision, $T_{collision}$ is equal.

Throughput of the system can be written as

(17) $$S=\frac{P_{s}E\left[L\right]}{P_{s}T_{success}+P_{c}T_{collision}+P_{f}Slotvalue}$$

where ***E***[***L***] is the average length of the packet. Delay is calculated as the sum of time for back-off and transmission time.

(18) $$E\left[D\right]=E\left[B\right]+E\left[T\right]=DIFS+T_{header}+T_{packet}+\sum_{i=0}^{W-1}\frac{i}{W}\left[\left(1-P_{b}\right)Slotvalue+P_{b}T\right]$$

## 5. Results and Discussion

Simulations in MATLAB are conducted to study the performance of the PCMAC. This protocol works with the MAC layer focusing on channel allocation without much delay for disseminating emergency messages. In VANET, on the occurrence of an event, an emergency packet must be generated and communicated to other vehicles within the region by the broadcast mode of communication through the control channel. MAC protocol, IEEE 802.11p, is used to broadcast the emergency packet over the control channel. The proposed protocol modifies IEEE 802.11p to adaptively tune the back-off according to the context of the generated packet.

### 5.1. Simulation Environment

A single lane road is considered with vehicles going in the same direction. All vehicles are equipped with an on-board unit register under a road-side unit, and they follow a single hop communication. The vehicles move as a platoon, and they know their position and velocity.

### 5.2. Communication Configuration

We have considered the communication range of each vehicle as 100 m. Vehicles can generate and broadcast messages. Scenarios where the vehicle has only an emergency packet, 20% emergency packets, 50% emergency packets, and 80% emergency packets are studied with the proposed protocol. The parameters used for simulating and studying the network are given in Table 4.

Time for the packet transmission is defined as a factor of length of the packet and data rate. The packet can either be an emergency packet or a non-emergency packet. Both the packets are of the size 100 bytes. Emergency and non-emergency packets belong to the safety category in IEEE 802.11p, and so the packet size is considered as 100 bytes. the time to transmit the header depends on the size of the MAC header and the data rate. The length of the header is taken as (L_h_) 50 bytes and the data rate is taken as 6 Mbps. In CSMA/CA protocol, the nodes sense the channel for a distributed inter frame space (DIFS) period before trying to attempt channel access. The time for sensing and transmission is divided into slots. Each slot is 13 µs. The value of DIFS is taken as 64 µs, as in IEEE 802.11p. To compare the performance of the proposed protocol and to demonstrate the need for considering the severity of data generation for improving the performance of IEEE 802.11p for forward collision warning, the following protocols with similar scenarios are studied.

- IEEE 802.11p: Simulations are performed to compare the performance of the fixed contention window with respect to the dynamic contention window for broadcast messages.
- R-MAC [27] is an adaptive MAC protocol for cooperative collision avoidance applications that consider the probability of an accident to happen. Time frame of R-MAC has three segments: the RSU broadcast, the TDMA for beacons, and the CSMA part for transmitting event messages. The number of CSMA slots depends on the probability of vehicle collisions that can happen in a platoon.
- Priority-based direction-aware MAC (PDMAC) [26] protocol is a cluster based V2V MAC with the objective of prioritizing warning message delivery in VANET. Protocol works with the TDMA approach considering the severity level, direction, and message type to prioritize the warning message. The scheme decreases the end-to-end delays and increases the network throughput.
- The following parameters are used to study the performance of the protocol.
- Delivery delay of emergency packet is the average time taken from the generation of a packet to its delivery.
- Packet delivery ratio (PDR) is defined as a fraction of the number of packets transmitted by the total number of packets generated during simulation.
- The relevance of location and velocity with respect to the event is studied with the parameter of how many nodes are affected if an accident occurs at a node. This parameter in turn shows the risk of having an accident in the area. It talks about the importance of making the message available to the nodes behind the vehicle in the accident.

### 5.3. Simulation Results

The protocol performance is investigated by simulating the scenario and comparing it with R-MAC protocol and traditional IEEE 802.11p. Figure 8 portrays the comparison of throughput performance of RMAC, PCMAC, and IEEE 802.11p. RMAC has higher throughput than PCMAC and IEEE 802.11p. R-MAC protocol has slots for beacons and adjusts the slots of CSMA for event messages based on the probability of the collision of nodes (accident). R-MAC protocol has TDMA slots for the beacons, and the event messages follow the contention scheme. The slots are already allocated for the beacons, and so the probability to transmit the packets without collision over the network is very high. Thus, RMAC has a higher throughput than the other two schemes. In PCMAC, both beacon and emergency packets contend to transmit the message. In IEEE 802.11p, the nodes are classified based on the traffic category in the node. Packets from nodes are prioritized for channel access using the transmission parameters AIFSN (arbitrary inter frame space number) and contention window (CW). However, the priority of a node with respect to its position and mobility is not considered in IEEE 802.11p. It uses a fixed contention window range. Therefore, as the number of vehicles increases, the throughput of the system deteriorates as many nodes may take the same waiting time to access the channel and may end up in a packet collision. PCMAC has better performance than IEEE 802.11p. It follows channel access based on the priority of the node and the packet. Each node will dynamically tune the contention window depending on the priority of the packet from the node at that instant. The priority is calculated as a function of average relative distance, average velocity, and impact of an event on the vehicles. This value is used to set the maximum contention window.

**Figure 9:**
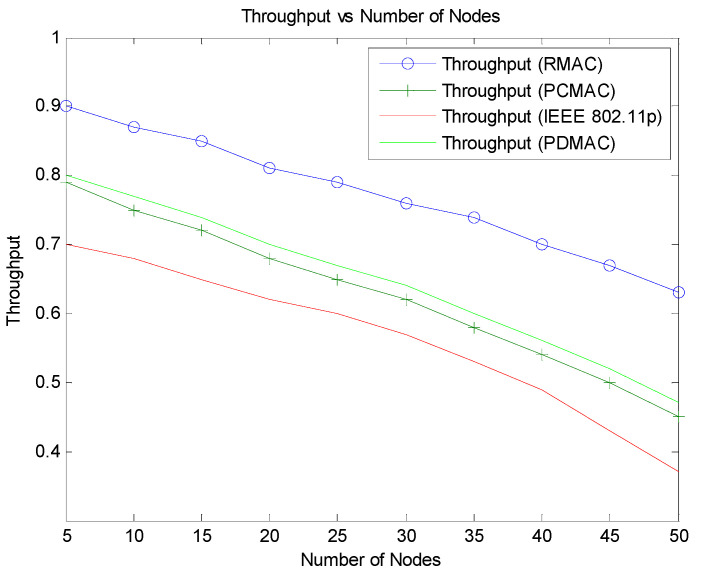
Throughput vs. number of nodes.

Figure 9 displays that the simulated results for throughput match with the analytical results of PCMAC. The graph was plotted for a varying number of nodes.

**Figure 10:**
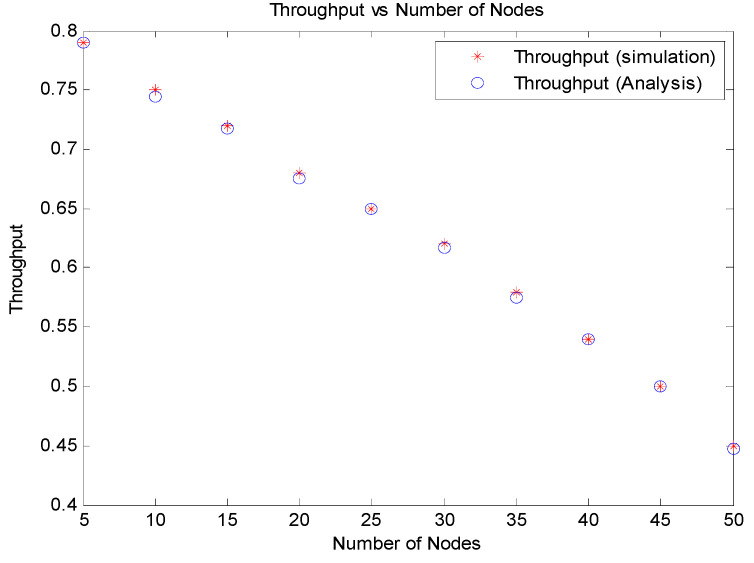
Throughput of PCMAC vs. number of nodes.

Figure 10 shows that PCMAC has a higher packet delivery ratio on a lesser number of emergency packets. We have studied the packet delivery ratio parameter for 20%, 50%, 80% and 100% of emergency packets. In PCMAC, both beacon and emergency packets contend to transmit the message. The emergency messages calculate the priority of the packet and contend for channel access with non-emergency messages that follow the IEEE 802.11p implementation. The emergency nodes have higher priority. Therefore, as the emergency messages in the network increases, the competition for channel access also increases, leading to a packet collision resulting in a low packet delivery ratio.

**Figure 11:**
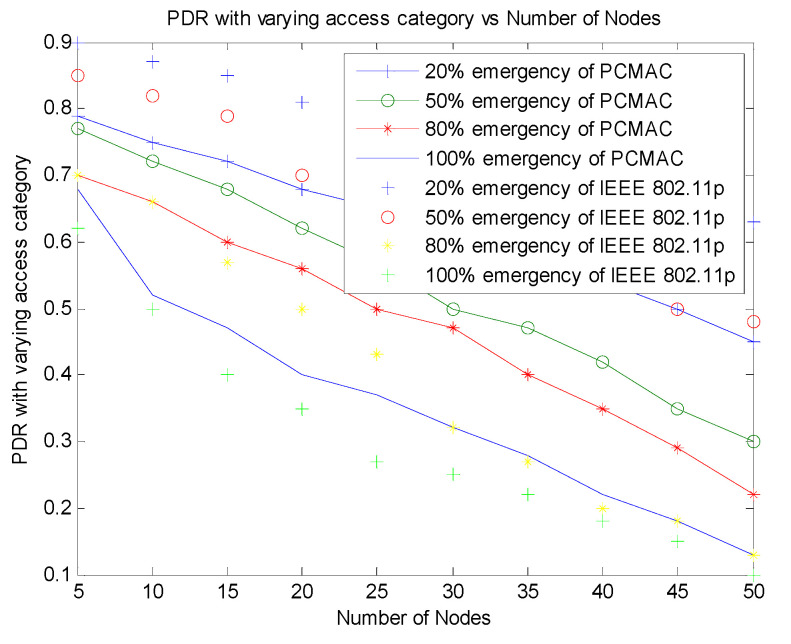
Comparison of PDR of PCMAC and RMAC with varying emergency packets.

Figure 11 displays the comparison of PDR with varying emergency packets. This plot shows that PCMAC has a better performance over RMAC and IEEE 802.11p with an increased number of emergency packets. In RMAC, the CSMA slots that are used for transmitting emergency packets are determined by identifying the probability of accidents. The beacons in RMAC are transmitted as per the slot allotted to the nodes. As the number of nodes increases, it becomes difficult to allocate disjoint slots for the nodes. The probability of accidents also increases, and therefore, the nodes contention to obtain channel access for emergency message transmission also increases, leading to a lower packet delivery ratio when the emergency packets are 80% and 100%.

**Figure 12:**
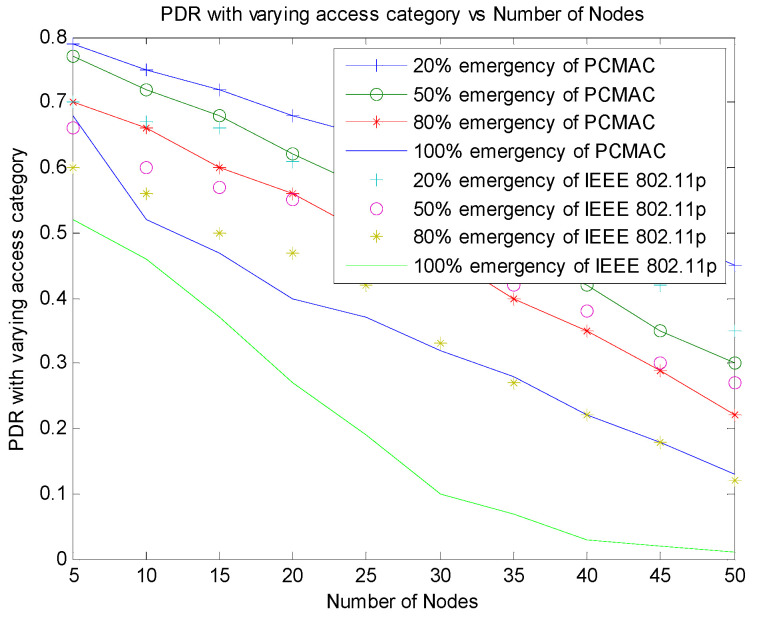
Comparison of PDR of PCMAC and IEEE 802.11p with varying emergency packets.

Figure 12 shows the comparison of PDR with varying emergency packets of PCMAC and IEEE 802.11p. In IEEE 802.11p, both beacons and emergency packets are given the same priority. Therefore, both beacons and emergency packets contend for channel access. When the number of nodes increases, the probability of emergency messages also increases, leading to more contention and more collisions of packets, and thus there is a lower packet delivery ratio. In PCMAC, the beacons and emergency packets are considered with different priority. The emergency packets are given channel access based on the context of information generated.

**Figure 13:**
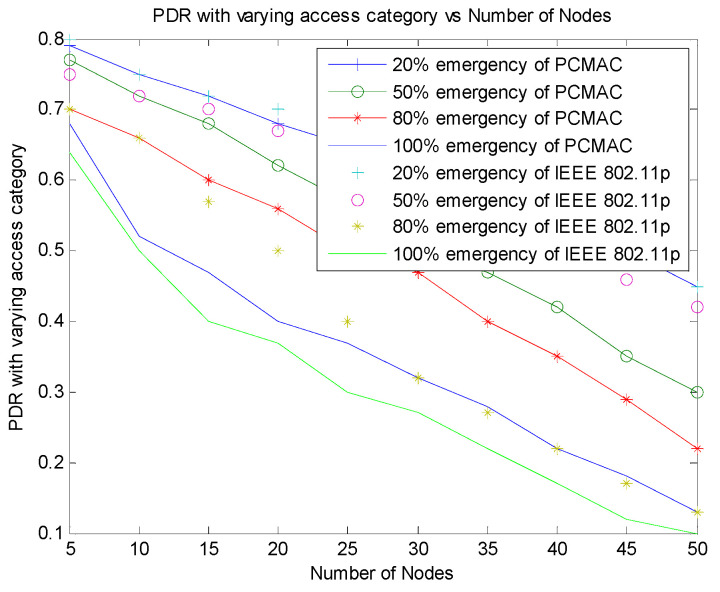
Comparison of PDR of PCMAC and PDCMAC with varying emergency packets.

Figure 13 shows the comparison of PDR with varying emergency packets of PCMAC and PDMAC. In PDMAC, works with a TDMA approach reserve slots based on the severity, message type, and direction. The severity depends on the probability of collision between the nodes. Therefore, as the number of emergency packets increases, the time slot reservation becomes complex, and thus lowers the packet delivery ratio. In PCMAC, the beacons and emergency packets are considered with different priority, and they both contend with each other based on the severity of the message. The emergency packets are given channel access based on the severity of the message generated.

**Figure 14:**
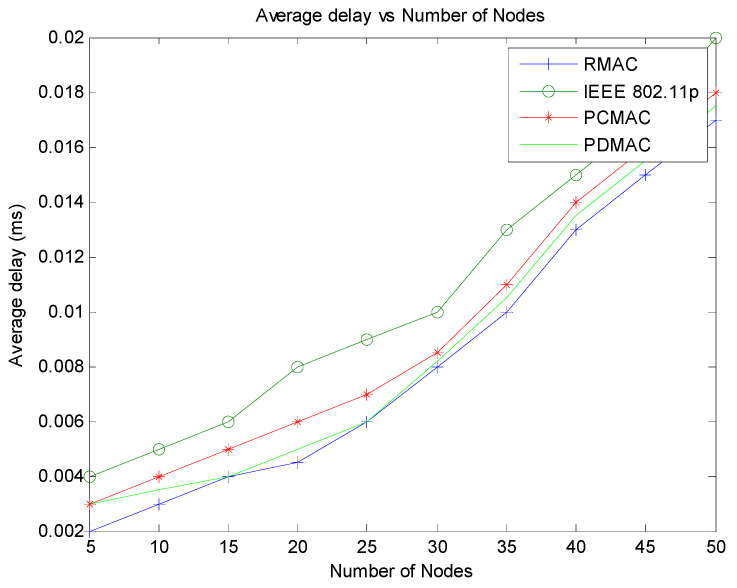
Average delay vs. number of nodes.

Figure 14 displays that the average delay of packet transmission is better for our protocol when compared with IEEE 802.11p. However, it is higher than the PDMAC and RMAC scheme. In R-MAC, the nodes have TDMA and CSMA slots in the frame. The system depends on RSU to obtain the probability of vehicles that can collide. This value is used to determine the CSMA slots, and the TDMA slots are used for beacon message transmission. The number of slots for beacon is already reserved. Only emergency packets follow the contention mechanism. Therefore, a few nodes compete for the channel with lesser collisions, so the delay in packet transmission is less for RMAC. In PDMAC, a priority-based TDMA scheme is used for slot reservation. Therefore, it has less delay when compared to PCMAC. However, as the number of emergency packets increases, in both RMAC as well as in PDMAC, the average delay increases. In PCMAC, all nodes compete for the channel, leading to a high probability for packet collision. This increases the average delay above R-MAC. For emergency messages, PCMAC calculates priority of the node at the instant. This value is mapped to the contention window. At the same time, the non-emergency messages use IEEE 802.11p implementation for channel access. Priority calculation helps in providing a service differentiation for different applications. IEEE 802.11p considers all the nodes with the same priority, and therefore, the chance of packet collision is more leading to a higher delay.

**Figure 15:**
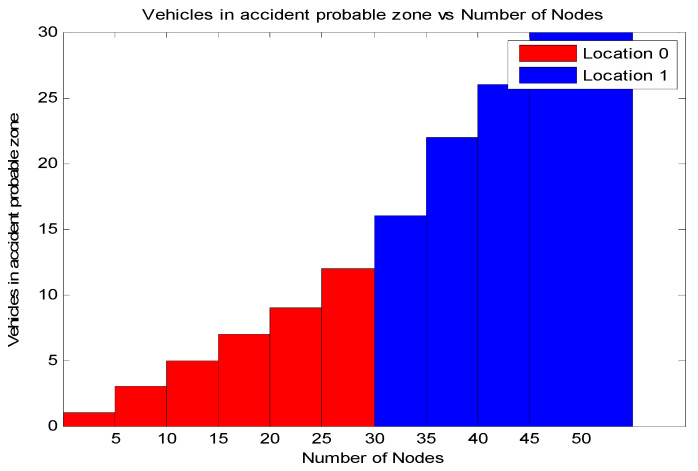
Number of nodes in a probable accident zone vs. number of nodes.

Figure 15 shows the importance of a location when giving priority for a message. Priority of the node at the instant depends on *Impact_i_*(*t*), average relative distance of nodes with respect to node_i_ at instant *t,* and average velocity of the platoon at instant *t*. *Impact_i_*(*t*) is defined as a factor of the number of nodes behind the node involved in the accident and the node density in the coverage. This gives an importance to the location of accident. Therefore, the importance of location is measured in terms of vehicles that follow the vehicles behind the node in the accident. The accident at a certain vehicle must be transmitted to the vehicles behind without much delay so as to avoid successive collisions. By receiving the message, the drivers can control the vehicle, avoiding successive collision. The priority of the packet from the node depends on the number of trailing vehicles of the node in the accident, the node’s relative velocity with respect to other nodes, and the average velocity of the platoon. This value is mapped to the contention window. The result displays that, as the number of vehicles increases, the risk in the probable accident zone also increases. The location of the accident zone in an RSU can be represented as below.

- Location 0: Starting location of the zone to R/2 where R is the range of the zone
- Location 1: Between R/2 to the end of the zone.

**Figure 16:**
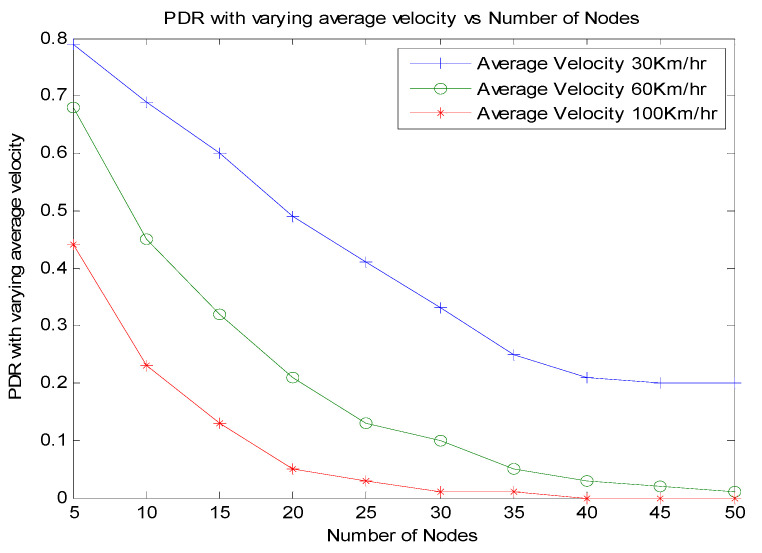
Effect of velocity on number of nodes in the zone.

Another factor that has an impact on the priority of an emergency packet from a node at an instant is the average velocity of the platoon. Figure 16 shows the effect of velocity, and this parameter is measured under three average velocities of the platoon. The average velocities are varied as 30 km/h, 60 km/h, and 100 km/h. The results show that the packet delivery ratio increases when the average velocity of the platoon is 30 km/h. As the average velocity of the platoon increases, the packet delivery ratio decreases. However, as the number of vehicles increase, the packet delivery ratio of the nodes will decrease.

**Figure 17:**
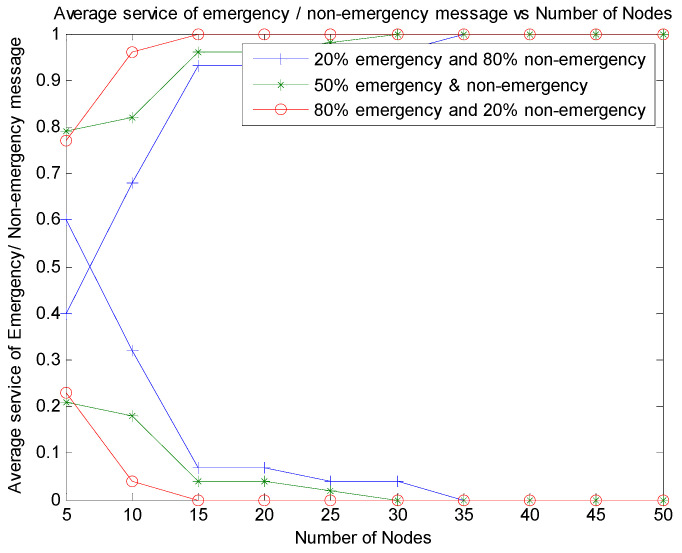
Average service of emergency message vs. number of nodes.

Figure 17 shows the average service processed for emergency messages. This parameter is measured as a factor of number of emergency messages processed out of the total number of emergency messages generated in the system. We have varied the emergency traffic as 20%, 50%, and 80% of the traffic from the network with nodes varying from 5 to 50. The results show that the emergency packets are serviced better than non-emergency packets, even if 80% of non- emergency packets exist in the system. When an equal number of emergency and non-emergency traffic exists, both types of traffic are initially considered. However, as the nodes increase, emergency packets are serviced more in all three scenarios.

## 6. Conclusions and Future Work

The proposed protocol works with the aim of enhancing the performance of IEEE 802.11p for collision awareness warning application in an intelligent transportation system. The emergency message should reach the drivers without much delay so that they can take the necessary steps to avoid accidents on the road. A mathematical modeling that supports the protocol for the broadcast communication of emergency vehicles is studied in this work. The protocol calculates the priority of the packet associated with a node and maps it to the contention window. The priority is calculated on the basis of the vehicle’s velocity, density, and location and the type of access category in it. The studies on the performance of the protocol shows that the protocol provides service differentiation for emergency messages when compared to non-emergency messages. It has a lesser delay in transmitting emergency packets with respect to the existing works. In PCMAC, we have considered only one platoon, where the vehicles move in a single lane under an RSU. However, in future works, we may consider connected platoons where the communication range of RSU may be extended considering the scenario of vehicles under different RSU.

## Figures and Tables

**Table 1:** Parameter value settings for access categories in IEEE 802.11p [4].

Access Category	VO	VI	BE	BK
AIFSN	2	3	6	9
CW_min_	3	7	15	15
CW_max_	7	15	1023	1023

**Table 2:** Classification of works under safety message dissemination.

Features	Method	Purpose	Disadvantages
Based on network statistics and density	[13,14,15,16,17,18,28] Adjust the contention window based on either the node density, transmission power or transmission probability.	Improves throughput, average packet delivery delay.	The mobility of the nodes is not taken into account. The nodes may leave the range if it has a higher velocity.
Based on mobility of nodes	[20,21,22,25] Adjust contention window based on the impact of velocity of the node.	Fair channel access, QoS.	It considers the velocity of the node, but the node density is another parameter that has a great impact on the quality of service of an application.
Based on the severity level of the message	[26,27,29,30,31] Nodes calculate priority of the message based on the severity of the message. The severity of the message is calculated based on the direction, location, access type, etc.	Improving QoS considering co-operative collision warning applications.	These schemes follow a hybrid approach, including both TDMA and CSMA schemes, to provide importance for both beacon and emergency schemes. Therefore, there is a need for time synchronization. A method for time synchronization is shown in [28]. The schemes follow a clustered approach.

**Table 3:** Notations and descriptions used in PCMAC.

Notations	Description
T	Every instant of time
v_i_(t)	Vehicle speed of node_i_ at instant *t*
e_i_(t)	Access category of node_i_ at instant *t*
i,j, etc.	ID
(x_r_,y_r_)	Range coordinates
(x_s_(t),y_s_(t))	Position coordinates of vehicle in accident
d_s_(t)	Distance to cover the range from the accident point
R_i_	Transmission range of node_i_
N	Number of nodes in the range
n_0_	Cardinality of the set of low priority nodes (non-emergency)
n_1_	Cardinality of the set of high priority nodes (emergency)
P_f_	Probability of channel to be free
P_b_	Probability of channel to be busy
P_s_	Probability of transmission to be successful
P_c_	Probability of collision
Impact_i_(t)	Importance of access category at node_i_ in the region at *t*th instant
AD_i_(t)	Average relative distance of nodes to node_i_ at instant *t*
AV_i_(t)	Average relative velocity of nodes to node_i_ at instant *t*
$\bar{V}$	Average velocity of the platoon
Priority(t)	Priority of nodei at instant *t*
T_transmit_	Time for transmitting a packet
CW_newmax_(t)	Upper limit of contention window for nodei at instant t. This is mapped to slots for defining a backoff value for node_i_ at instant *t*.
Backoff_i_(t)	Backoff value taken by nodei for communication at instant *t*. This is a random value between 0 and the maximum slots defined by UpBound_i_(t) of node_i_ at instant *t*
CW_max_[i]	Maximum contention window of the access category *i*
CW_min_[i]	Minimum contention window of the access category *i*

**Table 4:** Simulation Parameters.

Parameter	Values
Number of vehicles	5, 10, 15, 20, 25, 30, 35, 40, 45, 50
Speed range	10–100 km/h
simulation time	1000 s
Communication range	100 m
Emergency message size (L_p_)	100 bytes
Non-emergency size	100 bytes
MAC header (L_h_)	50 bytes
DIFS	64 µs
Slot value	13 µs
Data rate (data_rate)	6 Mbps
